# Review of En-Face Choroidal Imaging Using Spectral-Domain Optical Coherence Tomography

**Published:** 2013

**Authors:** Mahsa A. Sohrab, Amani A. Fawzi

**Affiliations:** Department of Ophthalmology Northwestern University, Feinberg School of Medicine, Chicago, IL, USA

**Keywords:** En-Face Choroidal Imaging, Spectral-Domain Optical Coherence Tomography, Choroidal Vasculature

## Abstract

Investigations of choroidal vasculature have been of particular interest given choroidal vascular dysfunction are thought to be related with a number pathologic conditions such as central serous chorioretinopathy and various forms of AMD, including polypoidal choroidal vasculopathy. On the other hand, en face imaging of the choroid allows an exceptional alternative to histopathologic evaluation of the choroid, and can be used to quantify choroidal vascular structures. Our former study verified differences in the macular choroid in AMD and control patients previously noted on histopathologic studies. The use of phase-resolved approaches in larger population longitudinal studies reveal the sequence of RPE and choroidal changes in the pathogenesis of various AMD subtypes, which cannot be done using histopathology. Issues with lateral resolution of the OCT system in measuring choriocapillaris size could be solved by incorporating the axial dimension of the choriocapillaris into choriocapilaris diameter assessment (assuming the choriocapillaris are round in vivo), and by correcting for anisometric pixel resolution. Forthcoming studies are required to determine whether areas of choriocapillaris correlate with areas of RPD lesions.

## INTRODUCTION

Advances in retinal imaging have led to improvements in our understanding of age-related macular degeneration (AMD), with current techniques allowing for improved visualization of the choroid. Various structural and functional approaches have been used to further elucidate the role of choroidal vasculature in the pathogenesis of retinal disease [1-8]. Earlier studies of the choroid relied on post-mortem tissue, with histopathologic comparisons of eyes from patients with early AMD to control eyes revealing a correlative increase in choriocapillaris loss and drusen density [1-3,5-8]. Studies of late-stage AMD showed constriction of remaining choriocapillaris and loss of normal choroidal vasculature underlying areas of intact RPE surrounding choroidal neovascular membranes as well as loss of choriocapillaris underlying retinal pigment epithelium (RPE) atrophy in atrophic AMD [4-5].

Subsequently, the development of indocyanine green angiography allowed for the in vivo study of choroidal vasculature [9], though it was limited in its ability to allow three-dimensional visualization of the choriocapillaris. Most recently, advances in spectral-domain optical coherence tomography (SD-OCT) have enabled more detailed visualization of the choroid through both enhanced depth and en face imaging, as well as correlations of indocyanine green angiography with en face imaging. Using enhanced depth imaging (EDI) [10-16], it has been shown that many patients with advanced AMD have significantly thinner choroids than their age-matched controls [10, 12-13]. Choroidal thickness has been shown to correlate highly with age, axial length, and refraction, and has been found to vary on a diurnal basis, suggesting that EDI can be an unreliable measurement of choroidal vasculature and underscoring the need to develop novel approaches for reliably assessing choroidal vascular health in vivo [16-18]. 

The use of en face imaging can provide detailed in vivo choroidal vascular reconstructions, which we have utilized to demonstrate the choroidal correlates of reticular pseudodrusen (RPD) [19] Furthermore, more recently we used en face choroidal imaging in a pilot study comparing control, in early AMD and RPD patients and found that choroidal vascular density in control eyes was comparable to control histopathologic studies, confirming the basic validity of this approach [20].

## HYPOTHESES

Studies of choroidal vasculature have always been of particular interest given choroidal vascular dysfunction are thought to be associated with many pathologic conditions like central serous chorioretinopathy and various forms of AMD, including polypoidal choroidal vasculopathy. The use of SD-OCT has enabled faster acquisition rates and high density three-dimensional data sets of the retina and choroid. En face OCT images have the flexibility to be reconstructed from this data set on a plane that follows the natural surface contour. In our case we have focused on examining datasets that follow the retinal pigment epithelium (RPE) and Bruch’s Membrane complex for our studies of the choroid. However, limitations to the use of current commercial en face imaging include the shadows created by more anterior structures such as the retinal blood vessels on deeper layers like the RPE and choroid, as well as the presence of uncorrected motion artifact [21-22]. 

Histopathologic studies of the choroid in patients with early AMD are very valuable, and have revealed that development of basal laminar and basal linear deposits is associated with areas of choriocapillaris atrophy and vascular dropout [1], [2], [4]. Furthermore, prior histopathologic studies have shown that choroidal vessel density and diameter both decrease as choroidal thickness decreases with age [1-5]. Given that remodeling of the choroid occurs with aging and in the course of AMD, a technique for in vivo en face study of the choroid would prove highly useful in allowing greater data acquisition as well as longitudinal studies to determine the sequence of changes as they relate to disease progression.

We hypothesized that en face SD-OCT slabs obtained through the choroid of patients with early AMD and control patients would correlate to the available histopathologic data quantifying vessel density and diameter in the three choroidal layers. We also hypothesized that alterations in the choroid were associated with the presence of RPD [19], originally identified on blue-light fundus photography and associated with loss of the small vessels of the middle choroidal layer and increased spacing between the large choroidal veins on histopathology [23].

## DISCUSSION

En face sections of varying choroidal thickness were first used to determine whether there were correlations between changes in choroidal vasculature and RPD lesions. OCT slabs were registered to infrared (IR) images and were manually registered to fundus imaging, revealing that individual reticular lesions tended to co-localize with choroidal stroma, closely abutting larger choroidal vessels. Areas of hyper-reflective sub-retinal deposits and associated outer retinal disruption frequently occurred directly adjacent to RPD lesions seen on IR imaging. When we overlayed en face OCT sections through the outer segment junction and sub-retinal space onto the IR images, we found that sub-retinal lesions are less prevalent than the RPD seen on IR images. Transitional overlays of varying opacities of choroidal en face images demonstrated that groups of reticular lesions closely followed the outline of large choroidal vessels [19].

After finding an association between choroidal vasculature and RPD, the next step was to hone our technique for more quantitative analysis of the choroidal layers in AMD, control, and RPD patients. We utilized two different approaches to evaluate choroidal vessel density, either using a full 6x6mm en-face scan, which provided an averaged macular vascular density and showed similar densities among the different groups, but suffered from segmentation artifact related to RPE curvature. Additional analysis using manually selected (500x500microns) regions showing more differences in the choriocapillaris density between the groups with surprisingly increased choriocapillaris vascular density in patients with RPD [20]. 

Qualitative evaluation showed non-uniform appearance of the vascular patterns suggesting patchy vascular loss, and examination of select regions of visible vasculature revealed an increased superficial choroidal vascular density in RPD eyes compared to early AMD eyes, after adjusting for age and gender (p=0.04) and choroidal thickness (p=0.004). The latter finding most likely resulted from the absence of choriocapillaris on all of the RPD eyes, visible as lack of “granular appearing” choriocapillaris in the sub-RPE region on SD-OCT cross-sections and lack of the regular “honeycomb” pattern on most superficial en face scans of those eyes as compared to early AMD and control eyes. Furthermore, given the limited resolution of the en face approach, it is likely that that residual “ghost” or non-perfused choriocapillaris, which are likely less than a horizontal pixel (11 microns), are under-estimated [20]. 

Similar to histopathologic findings of choriocapillaris atrophy and vascular dropout in areas of basal laminar and basal linear deposits [1-8], SD-OCT cross-section scans showed that less than half of the eyes with early AMD had visible choriocapillaris. Early AMD eyes also had significantly decreased choriocapillaris vessel density as compared to controls (79.7% versus 86.7%, p=0.04), as well as slightly reduced choriocapillaris diameter, though the latter was not statistically significant (4.19 versus 4.49 pixels, respectively) [20]. Our study validated histopathologic measurements of vessel density, supporting the utility of OCT scan as a reliable tool for quantitative choroidal vascular mapping in vivo. Control eyes had an overall choroidal vessel density (6x6mm) of 78.5%, closely paralleling 79.6% reported in histopathologic studies. Vessel diameters found in this study averaged 40-50 microns for the choriocapillaris compared to 100 and 200 microns for medium and large choroidal vessels, respectively, also consistent with histopathologic evidence in the posterior pole, though our approach likely leads to overestimation of the choriocapillaris size. Choriocapillaris size assessment with en face imaging is limited by the lateral resolution of the OCT system, which is 15 microns, as compared to the average macular choriocapillaris size on histopathology, which is about 20 microns [20]. 

We found that patients with severely thin choroids (0 to 100 microns) had a lower average vessel density in the innermost choroidal layers as compared to thicker choroids (300 to 400 microns), in whom vessel density was similar between the layers, also consistent with previous histopathologic evidence of decreased choroidal vessel density and diameter with decreased choroidal thickness [1-5]. Our data suggests that choroidal thinning is associated with highest decrease in vessel density of the inner layer of the choroidal vasculature with less effect on the outer vasculature. The proximity of the choriocapillaris to the RPE allows a high oxygen supply to the highly metabolic outer retina, and the effect of decreased density of choriocapillaris in the setting of early AMD can have important implications on disease progression [20]. 

Our results confirmed that the choroid becomes thinner with increasing age irrespective of disease status, and that this change is more pronounced in RPD patients as compared to those with early AMD or controls. Further analysis of our results demonstrated that, while overall choroidal thickness decreases in both men and women with age, choroid tends to be relatively thinner in younger women and older men, consistent with a recent study noting gender differences in choroidal thickness [24], and suggesting that age and gender may play a more important role in determining choroidal thickness than the underlying disease processes.

Limitations of our approach include the use of arbitrary cut-offs to separate vessel from stroma, as compared to histopathology, where more specific vascular labeling is possible. However, the pixel intensity cut-offs were standardized and applied to all groups allowing these comparisons. Furthermore, histopathology can differentiate between healthy and ghost choriocapillaris where structural imaging cannot, an issue that may be addressed with newer technologies such as phase-resolved OCT [25].

## CONCLUSION

En face imaging of the choroid allows an excellent alternative to histopathologic study of the choroid, and can be used to quantify choroidal vascular structures in the aging population in vivo. Our study verified differences in the macular choroid in AMD and control patients previously noted on histopathologic studies. The use of phase-resolved approaches in larger population longitudinal studies will help elucidate the sequence of RPE and choroidal changes in the pathogenesis of various AMD subtypes, which cannot be done using histopathology. Issues with lateral resolution of the OCT system in measuring choriocapillaris size could be solved by incorporating the axial dimension of the choriocapillaris (from cross-sectional b-scans) into choriocapilaris diameter assessment (assuming the choriocapillaris are round in vivo), and by correcting for anisometric pixel resolution. Future studies are needed to determine whether areas of choriocapillaris correlate with areas of RPD lesions.

## DISCLOSURE

Conflicts of Interest: None declared.
